# Case report of early signs of aortic stenosis decompensation detected via ambient sensor-derived digital biomarkers

**DOI:** 10.1093/ehjcr/ytae655

**Published:** 2024-12-11

**Authors:** Nisha Arenja, Narayan Schütz, Philipp Buluschek, Tobias Nef, Hugo Saner

**Affiliations:** Solothurner Spitäler AG, Schöngrünstrasse 36A, 4500 Solothurn, Switzerland; Stanford University, Stanford Way, Stanford, CA 94305, USA; EPFL Innovation Park, Building D, 1015 Lausanne, Switzerland; ARTORG Center for Biomedical Engineering Research, Freiburgstrasse 3, 3010 Bern, Switzerland; ARTORG Center for Biomedical Engineering Research, Freiburgstrasse 3, 3010 Bern, Switzerland

**Keywords:** Heart failure, Aortic stenosis, Home monitoring, Ambient sensor system, Case report

## Abstract

**Background:**

Aortic stenosis is a progressive condition with a grim prognosis, underscoring the importance of timely intervention to prevent decompensation. Home monitoring systems, particularly those utilizing ambient sensors, offer promise in detecting early signs of deterioration.

**Case summary:**

We present the case of an 89-year-old woman who was asymptomatic but monitored using such a system prior to experiencing acute decompensation. Key clinical indicators, including increased night-time heart rate, respiration rate, toss and turns in bed, and nocturia, were observed several months before hospitalization. Additionally, reduced physical activity and increased toilet visits were noted.

**Discussion:**

These findings highlight the potential of ambient sensor systems in identifying pre-clinical stages of cardiac decompensation, especially in severe aortic stenosis cases. Integrating ambient sensor systems into routine clinical practice holds promise for enhancing proactive management strategies and reducing adverse outcomes associated with cardiovascular disease progression.

**Learning points**

- Recognizing symptoms and manifestations of severe aortic stenosis is vital for determining the need for interventions such as transcatheter aortic valve implantation (TAVI) or surgical aortic valve replacement.
- Home monitoring systems can capture early important clinical changes, which may indicate heart failure (HF) decompensation.
- Utilizing digital biomarkers from non-intrusive ambient sensor systems can provide early detection of HF decompensation, particularly in patients with severe aortic stenosis.
- Timely intervention with procedures like TAVI is crucial to mitigate risks associated with emergency procedures and reduce mortality rates.

## Introduction

Aortic stenosis presents a grave prognosis as it progresses over time. Recognizing early signs of its advancement is pivotal to prevent heart failure (HF) decompensation and enable timely intervention, such as transcatheter aortic valve implantation (TAVI). Notably, a significant portion of TAVI procedures are conducted on patients experiencing acute symptoms, necessitating urgent valve replacement. However, this subgroup often faces more complicated post-interventional courses and higher mortality rates compared with stable patients with milder symptom.^1^ Hence, optimizing the timing of aortic valve replacement (AVR) is paramount.^2^

In recent years, there has been a growing trend towards employing home monitoring systems, particularly multi-modal ambient sensor systems, to surveil seniors in their residences. These systems serve to detect emergency situations and to monitor digital biomarkers indicative of clinically relevant health issues over extended time periods.^3,4^ Notably, clinical indicators of HF decompensation, such as elevated heart and respiration rates, decreased physical activity, reduced gait speed, increased nocturnal toileting, and deteriorating sleep quality, hold significant potential for detection by non-intrusive, contactless ambient sensor systems.^5^ Recognizing negative changes in these parameters could aid in preventing further deterioration and subsequent hospitalization due to HF decompensation.^6^

## Summary figure

**Table:** 

Date	Event
May 2022	Start heart rate increase/night in bed
August 2022	Start increase toss and turns/night in bed
August 2022	Start increase number of toilet visits at night
14 December 2022	Escalating breathing difficulties
16 December 2022	Emergency visit to the family doctor and hospitalization due to decompensated heart failure
21 December 2022	Diagnosis of severe aortic stenosis
22 December 2022	Successful TAVI procedure
	Post-interventional complications
17 January 2023	Discharged home

## Case summary

An 89-year-old woman, a retired home economics teacher, resides independently in a three-room apartment within a senior’s residence. Mentally sharp, she remains engaged in household activities, particularly baking and knitting wool socks for those in need.

Two years preceding her cardiac incident, she and her daughter opted to install the DomoCare 360° safety ambient sensor system (DomoHealth, Lausanne, Switzerland) to enhance home safety. Prior to the index event, she reported good health, with no specific medical diagnoses except for occasional mild breathing issues attributed to ageing. While previously using midazolam for sleep problems, it was later replaced by diphenhydramine. Apart from that, she was not taking any long-term medication. Subtle changes, including propping up her headboard for easier breathing and increased night-time bathroom visits, were noted in the months leading up to her acute HF decompensation. Despite these, she did not perceive significant health decline and seldom visited her family doctor.

On 14 December 2022, she expressed escalating breathing difficulties during a visit from her daughter, prompting her daughter to advise a prompt visit to the family doctor. By 16 December 2022, her breathing problems intensified, leading to an urgent appointment arranged at the family doctor’s office. The family doctor diagnosed acute HF decompensation, likely attributed to atrial fibrillation, which was previously unknown instead of known and promptly referred her to a nearby secondary care hospital.

*Physical examination at hospital entry* revealed shortness of breath, Glasgow Coma Scale 15, blood pressure 155/100 mm Hg, heart rate (HR) 117 b.p.m., SO_2_ 95%, and suspected bilateral pleural effusion.

*Ultrasound examination of the thorax* confirmed bilateral pleural effusion.

*Chest X*-ray and *computer tomography thorax* showed only minimal residual pleural effusion after drainage, no signs of pulmonary embolism, no signs of right HF, and no conclusive signs of cardiac decompensation.

*Electrocardiogram findings revealed s*mall complex tachycardia, HR 117 b.p.m., Sokolow index left >3.5 mm, and no ST-elevation.

*Transthoracic echocardiography findings included* concentric hypertrophy of the left ventricle with normal systolic function (left ventricular ejection fraction 53%), whereas diastolic function could not be assessed conclusively. Dilatation of the left atrium. Severe degenerative aortic stenosis (dp mean of 42 mm Hg and calculated valve opening surface of 0.4 cm^2^), severe mitral insufficiency, normal dimensions and function of the right ventricle, systolic pulmonary artery pressure estimated at 44 mm Hg. No pericardial effusion.

*Laboratory findings were as follows* (normal ranges): natrium 141 (135–145 mmol/L), potassium 3.6 (3.5–4.8 mmol/L), calcium 2.11 (2.20–2.55 mmol/L), creatinine 51 (44–80 μmol/L), N-terminal pro-brain natriuretic peptide 6101 (<125 ng/L). C-reactive protein, blood glucose, and thyroid stimulating hormone were all within the normal range.

In summary, extensive evaluation revealed severe aortic stenosis accompanied by mitral insufficiency and pulmonary congestion.

Following initial management of the HF decompensation including successful puncture and drainage of the bilateral pleural effusions, she was transferred to a primary care hospital for TAVI. A cardiological assessment on 21 December 2022 confirmed severe aortic stenosis (mean gradient of 42 mmHg and valve area of 0.4 cm^2^), severe secondary mitral insufficiency, mild coronary artery disease without significant stenosis, normal left ventricular function with an ejection fraction of 55%, Society Thoracic of Surgeons-Score for AVR of 7.1%, and persistent atrial fibrillation.

On 22 December 2022, the TAVI procedure was successfully performed without complications. Three days later, due to a third-degree atrioventricular block, a permanent pacemaker was implanted with a ventricular pacing rate of 70 b.p.m., which resulted in persistent atrial fibrillation and a constant pacemaker rhythm. Additionally, she experienced post-interventional complications including anaemia requiring blood transfusion and hypo-albuminuria managed with protein-rich nutrition.

Medication at discharge included verapamil hydrochloride 120 mg 1-0-1, torasemide 10 mg 1-0-0, fragmin 5000 I.E. 0-0-1, magnesium 300 mg 1-0-0, and metamizole 500 mg 2-2-1.

Subsequently, the patient underwent residential cardiac rehabilitation, achieving a satisfactory recovery over a 3-week period. By 17 January 2023, she was discharged home, able to resume a relatively independent lifestyle. This 89-year-old lady has been relatively frail before the event, and the increase in physical fitness has been limited after rehabilitation.

Physical activity within the apartment and toilet visits were assessed using a commercially available passive infra-red (PIR) motion sensing system from DomoHealth SA (Lausanne, Switzerland). This system comprised five PIR motion sensor units and two magnetic door sensors wirelessly connected to a base unit. The motion sensors captured movement in equipped rooms at a frequency of once every 2 s (0.5 Hz), all communicating via the Zig Bee protocol to the base unit, which then transmitted data to the cloud in real time using the Global System for Mobile Communications (GSM) network. Each of the subject’s kitchen, living room, entrance, bedroom, and bathroom was equipped with one PIR sensor. Motion signals were collected room by room and amalgamated to quantify total activity in the apartment. Normalized daily PIR sensor activity denoted the duration of PIR sensors registered activity at home normalized by the total time spent at home for a given day, thereby accounting for time spent outside the home. A bathroom visit was identified if at least one sensor firing in the bathroom was recorded within a 30-min window. Night-time was defined as the period from 8 p.m. to 6 a.m. local time, aligning with the patient’s daily activity patterns. Data processing and visualizations were executed using Python programming language version 3.6.

**Figure 1:**
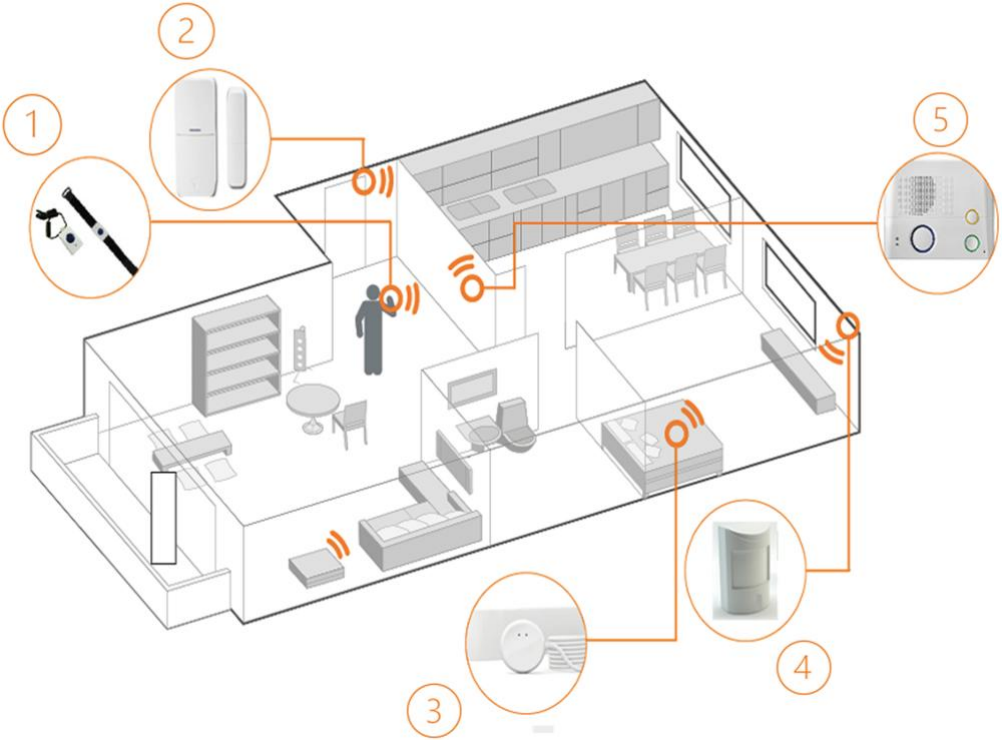
Ambient sensor system from DomoHealth® as used for this case report. Digital biomarkers are collected from passive infra-red motion sensors in each room of the apartment, from a door sensor to monitor home exits and from an Emfit QS bed sensor, which is placed under the mattress of the patient and collecting data on heart rate, respiration rate, toss and turns in bed, and sleep parameters. The system is designed for persons living alone in their home to provide preventive and emergency information. Data are transmitted to an interphone and from there to the system server and from there to the 24/7 emergency call centre, to medical professionals such as home care nurses or family doctors and to care providers including family members. Upon request, periodical health reports are created and sent to the patient and/or the care givers including information about physical activity, sleep, and social contacts to increase the awareness for health issues and to support healthy lifestyle behaviour. Graphical representation of the ambient sensor system used in this study [1. alarm button, 2. door sensor, 3. Emfit QS bed sensor, 4. PIR motion sensor in each room (including bathroom), and 5. interphone].^4^

Heart rate, respiration rate, and toss-and-turn counts during sleep were captured using the EMFIT QS device (Emfit Ltd., Jyväskylä, Finland).^7^ This contact-free piezoelectric sensor, placed beneath the subject’s mattress, utilized thin quasi-piezoelectric films to detect subtle pressure changes generated by the beating heart. Transmitting data to the cloud in real time via local WiFi and subsequently the GSM network, the device extracted vital signs including HR, respiration rate, HR variability, movements in bed, sleep duration, and sleep onset delay. Recent findings indicate its accuracy in measuring HR and respiration rate (*Figure 1*).^8^

In our case, the average night-time respiration rate decreased from 16–17/min before the acute event to 14/min after TAVI. The average night-time HR increased from 66 b.p.m. over 7 months to 75 b.p.m. before the acute event, subsequently decreasing to 60–72 b.p.m. after TAVI. Toss-and-turn counts, indicative of discomfort, increased by over 30% 6 weeks before the acute event and decreased significantly post-TAVI. The number of nightly toilet visits rose from 2.2 to 7 before cardiac decompensation, declining to 3/min after TAVI.

## Discussion

We present the first case utilizing digital biomarkers derived from a non-intrusive ambient sensor system, tracked over several months preceding acute HF decompensation of aortic stenosis in an ostensibly asymptomatic 89-year-old woman. Timely intervention with TAVI is imperative to facilitate intervention under stable conditions, mitigating the elevated risks associated with emergency procedures and reducing mortality rates. Hence, there is a pressing need to avert aortic stenosis decompensation, both clinically and economically.

**Figure 2:**
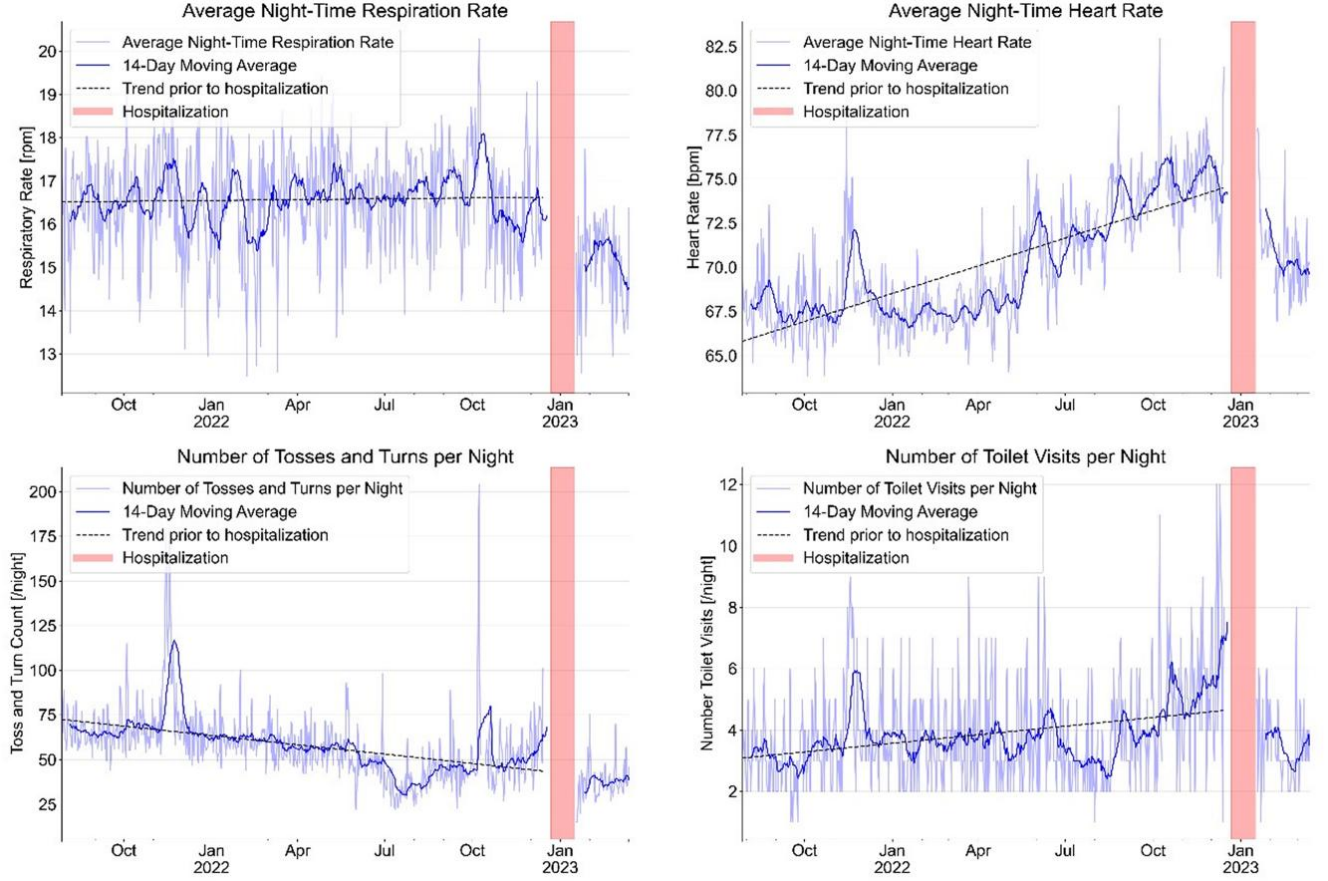
Evolution of sensor signals from a combined ambient passive infra-red motion sensor system to monitor activity and sleep from passive infra-red motion sensor system (normalized units) in combination with an EMFIT QS bed sensor device over a period of 1 year. The combination of signals from the various sensors before hospitalization indicates first signs of increasing heart failure decompensation several months before the index event in December 2022. After hospital discharge, sensor signals indicate improvement of all parameters.

Indications for TAVI or surgical AVR hinge on the severity of aortic stenosis and the presence or absence of symptoms.^1,2^ Classic symptoms of severe aortic stenosis include chest pain, fainting, shortness of breath, fatigue, and palpitations, although these may be overlooked or attributed to ageing in the elderly. Our ambient sensor system began detecting clinical signs of HF decompensation more than 3 months before hospitalization, with bed sensor data revealing escalating nocturnal HR and increased respiration rate persisting for over a year prior to acute HF decompensation. A surge in nocturnal bed exits and toilet visits further signalled cardiac decompensation (*Figure 2*).^8^

Our findings underscore the potential of home monitoring signals from non-intrusive ambient sensor systems to detect early signs of HF decompensation, particularly in severe aortic stenosis. Clinical manifestations such as nocturnal shortness of breath, changes in HR, increased nocturia, restless sleep, and reduced physical activity can be easily captured by these systems, generating valuable health insights through signal combination.

Remote monitoring with cardiac implantable electronic devices (CIEDs) is nowadays standard in many centres for rhythm monitoring, control of device function, and therefore for safety reasons.^9^ In the Mid-HEFT trial, investigating the OptiVolTM technology (Medtronic, Ireland), changes in intrathoracic impedance preceded the onset of HF symptoms by an average of 15 days.^10^ In a retrospective analysis of 1719 patients, Zile *et al*.^11^ showed the usefulness of intrathoracic impedance measurements for risk stratification of long-term HF hospitalizations.^11^ In our case report, the patient received a pacemaker implantation after TAVI. The additional monitoring function can be combined with the ambient sensor to increase the diagnostic value. Remote monitoring systems can be useful for more than detecting HF decompensation. Studies have shown that an advanced algorithm using the transthoracic impedance of the CIED can be used to detect sleep apnoea.^12,13^ In our patient with a permanent third-degree atrioventricular block after TAVI, there was a constant pacing rate of 70 b.p.m., which corresponds to a mean HR between 69 and 72 b.p.m. as indicated by the Emfit QS bed sensor.

While individual digital biomarkers may indicate diverse health issues, their amalgamation facilitates the identification of HF decompensation. Although these combinations may not always provide a definitive diagnosis, they signal significant health issues warranting prompt healthcare professional intervention. Early alerts to healthcare providers hold promise in facilitating timely interventions, thereby minimizing complications and mortality rates. In addition, the improvement in cardiorespiratory fitness, e.g. through cardiovascular rehabilitation, can be monitored and adjusted if necessary.^14^ In our case, we cannot exclude an impact of fitness improvement on the improvement of presented parameters.

The average respiration rate of the patient was 16.5/min with minor daily deviations over the whole year before the event and decreased to 14/min after TAVI. However, differences are small and not significant. We can only speculate that this could be an indicator for a lack of significant pulmonary congestion before the acute decompensation. The constant increase of the mean HR at night from 66 to 74 b.p.m. before TAVI can be considered as a significant indicator for a health issue although not specific for a primary cardiac problem. Movements in bed described as toss and turns have been relatively stable between October and June followed by a considerable decrease in July and August, which was most probably due to the introduction of a sleep medication at this time. The increase of toss and turns in bed from August 2022 to January 2023 from an average of 29 to 68 is highly significant. As we have previously shown, movements in bed are a most important sleep parameter, and an increase in the number of toss and turns in bed over time is a strong indicator for health issues.^4^ A constant increase of toilet visits at night from a mean of three times per night to seven times per night in the absence of a urinary problem can also be considered as a relatively strong diagnostic criterion for increasing HF decompensation.

As this is the very first case report of ambient sensor signals in a patient with severe aortic stenosis necessitating TAVI, we cannot provide any information in regard to cut-off points as indicators for the necessity of a consultation. However, ambient sensor data from other patients with HF decompensation indicating HR and respiration rate increase before decompensation led to the decision to search for such cut-off points in a prospective study including elderly HF patients after decompensation, for which the research protocol has been recently published.^15^ Prospective studies are warranted to fully explore the clinical potential of these findings in HF patients’ contexts.

## Data Availability

The data underlying this article will be shared on reasonable request to the corresponding author.

## Lead author biography

Nisha Arenja is a senior cardiologist and works at Solothurner Spitäler AG. She specializes in cardiac imaging and heart failure, which is also the focus of her research.

**Consent:** The authors confirm that written consent for submission and publication of this case report associated text has been obtained from the patient in line with COPE guidelines.

**Funding:** None declared.
